# The Effect of the Level of Goat Liver Addition to Goat Minced Meat on the Near-Infrared Spectra, Colour, and Shelf Life of Samples

**DOI:** 10.3390/foods14081430

**Published:** 2025-04-21

**Authors:** Louwrens Christiaan Hoffman, Wencong Wu, Shuxin Zhang, Michel Beya, Daniel Cozzolino

**Affiliations:** Centre for Nutrition and Food Sciences (CNAFS), Queensland Alliance for Agriculture and Food Innovation (QAAFI), The University of Queensland, Brisbane, QLD 4072, Australia; louwrens.hoffman@uq.edu.au (L.C.H.); shuxin.zhang1@uq.net.au (S.Z.);

**Keywords:** liver, minced meat, CIELab, pH, NIR, goat

## Abstract

This study aimed to evaluate the utilisation of near-infrared (NIR) spectroscopy combined with chemometric techniques to identify the addition of goat liver to goat minced meat and to monitor the shelf life of the samples up to 8 days of storage. Mix samples were created by adding goat liver to goat meat in different ratios (0%, 2%, 4%, 6%, and 8% w/w), and after mincing, the samples were stored under chilled (2–4 °C) conditions for 8 days. The NIR spectra, CIELab parameters, and pH of the mixture samples were collected at the start of the study and after 2, 4, 6, and 8 days of storage. The mince became darker with the increase in days of storage, while the pH value was not affected by days of storage. Partial least squares (PLS) regression was used to develop calibration models for the CIELab parameters to predict the level of liver addition to minced meat and to predict days of storage. The standard error in cross-validation (SECV) and the coefficient of determination in cross-validation (R^2^_cv_) were 0.10 (SECV: 3.3), 0.63 (SECV: 1.5), and 0.60 (SECV: 0.90) for L*, a*, and b*, respectively. The R^2^_CV_ and SECV were 0.32 (SECV: 2.4%) and 0.92 (SECV: 0.98 days) to predict the level of liver addition to minced meat and days of storage, respectively. The NIR calibration models developed to predict the CIELab parameters and level of addition of liver to minced meat were inadequate for predicting new samples. On the other hand, the PLS models developed could predict the days of storage, R^2^_cv_ 0.92 (SECV: 0.98 days). Compared with traditional methods such as CIELab or pH measurements, NIR spectroscopy can yield results more rapidly. However, the variability in the data set should be increased to allow the development of more reliable models.

## 1. Introduction

Minced and processed meat products are acquired in most parts of the world by consumers. These products contribute a significant proportion of the available portfolio of meat companies [1]. Furthermore, the minced meat market tends to increase annually due to better accessibility and price for consumers [1].

The utilization of offal and other animal byproducts has been proposed to reduce waste as well as to add compounds or nutrients of functional value to minced meats from different animal species [2,3,4]. Offal is defined as the internal organs and entrails of a butchered animal, which include the kidneys, tongue, brains, feet, stomach, heart, liver, and lungs [3]. Offal is also considered a delicacy in different countries. However, labelling schemes used for minced meat restrict products containing meat cuts and tissues where maximum limits of natural fat and connective tissue are allowed to be added [3]. Consequently, the addition and presence of non-meat offal cuts or ingredients must be declared on the end-product label [5]. It is well known that offal tissues carry a lower market value than other meat cuts and tissues of low value in some global markets. However, due to significant differences in price compared with the meat skeletal tissue, adding low-cost offal cuts to minced or processed meat products is a common practice in the meat supply and value chain of different species in different countries [3,5]. Regardless, including offal ingredients in any meat product in many countries must be declared on the label [5].

Among the different offal cuts, liver is a tasty and nutrient-dense ingredient frequently included in stuffing recipes in combination with varying cuts of meat from different species [6]. However, the stability (e.g., oxidation and shelf life) of the meat mince and mixture products (e.g., minced meat plus offal) can be influenced by the high concentration of iron, lipids, and fatty acids and other compounds or nutrients of high functionality [1,4,6]. In this context, adding different liver levels may impact goat minced meat stability and shelf life [7]. It has also been reported that adding offal (e.g., liver) to minced meat can greatly enhance its nutritional value and functional properties [7,8]. Adding offal (e.g., liver) to minced meat can increase the amount of protein, iron, and vitamins A and B12 [7,8]. However, high amounts of liver added to goat minced meat might cause negative effects, such as enhancing strong flavour notes that some consumers might dislike [9,10].

Iron is a pro-oxidant and can actively be involved in the chemical and biochemical reactions associated with the oxidation of lipids, which might cause meat and meat products to deteriorate and become rancid [11,12,13]. Furthermore, the colour of the meat, texture, and nutritional value might also change due to oxidation [11,12,13]. To guarantee the best stability and shelf life, it is crucial to properly balance the liver content when mixed with minced meat [11,12]. According to published studies, adding high proportions of the liver to minced meat or stuffing combinations can assist in maintaining the integrity of the end product [11,12,13]. Adding offal to minced meats can also influence the storage conditions, packing, processing, and, overall, the shelf life and stability of meat products [11,12,13]. Furthermore, the shelf life of goat products can be improved by using proper refrigeration (e.g., low temperature and humidity), by using vacuum sealing packaging, or by adding ingredients such as offal products [11,12,13].

It is well known that after mincing, the morphological characteristics of meat tissues are drastically altered or destroyed [5,14]. Therefore, it is challenging for the consumer to identify the adulteration with or the addition of other meat species of low value or offal cuts when purchasing minced meat [5,14]. The European horsemeat scandal in 2013 showed that the vulnerability of the meat supply and value chains is still under threat due to issues such as meat adulteration or fraud [5,14]. For example, substituting one animal species with a cheaper alternative is the most exploited and well-known form of meat fraud [5,14]. However, other options are utilised in the meat supply and value chain to achieve adulteration or fraud, including misleading information about the country of origin, sex, type of cuts utilised, the breed, or age of the animal or even making false claims about the processing or treatment and non-meat ingredient additions [5,14,15]. More importantly, adulteration and fraud in the food value chain not only affect the safety of the food or the health of the consumers but also result in critical economic losses for society and the food industry.

While several methods and technologies have been evaluated and implemented for their ability to determine or monitor the level of adulteration or fraud in meat products with offal cuts, no single method has been recognised and validated as a reliable test to support legislative requirements [5,14,15]. In this context, a wide range of technologies and methods can be used alone or in combination to target issues associated with food adulteration. These methods might vary from determining the sample’s elemental composition to measuring specific molecules or compounds such as peptide markers [5]. Overall, all methods available provide a different level of accuracy or applicability. However, all of them strive to contribute to determining the presence and level of the offal tissues present in the sample to support the safety and integrity of the food chain [5,15]. With the current developments in modern instrumental analytical technologies and the integration of chemometrics and machine learning (ML) methods, continuous innovations and improvements have appeared [5,15]. One such technique is near-infrared (NIR) spectroscopy. This technology has benefits for improving detection efficiency, is highly portable, and is one of the most widely used technologies in detecting meat products’ safety [16,17,18,19]. Ultimately, NIR spectroscopy has contributed to improving the safety of meat products and safeguarding the ultimate rights and interests of consumers and their health issues [16,17,18,19].

As stated, NIR spectroscopy has been used to evaluate, monitor, and assure the safety of meat and meat products [16,17,18,19]. Several examples have demonstrated the advantages of NIR spectroscopy in measuring chemical properties in meat, assessing and tracing the authenticity of meat and meat products, and other applications [16,17,18,19,20,21,22,23,24,25,26]. Recently, NIR spectroscopy was reported to either assess or monitor the addition of offal to different meat species. Examples of these applications include the identification of adulteration of beef burgers with offal using NIR spectroscopy [27], the assessment of the adulteration of pork meat with offal using hyperspectral imaging [28], and the use of Fourier transform mid-infrared to trace the addition of offal to beef meat [15].

This study aimed to evaluate the utilisation of near-infrared (NIR) spectroscopy combined with chemometric techniques to identify the addition of different levels of goat liver to goat minced meat. Furthermore, NIR spectroscopy was also used to monitor the shelf life of the mix samples up to 8 days of storage.

## 2. Materials and Methods

### 2.1. Samples and Experimental Design

**Figure 1:**
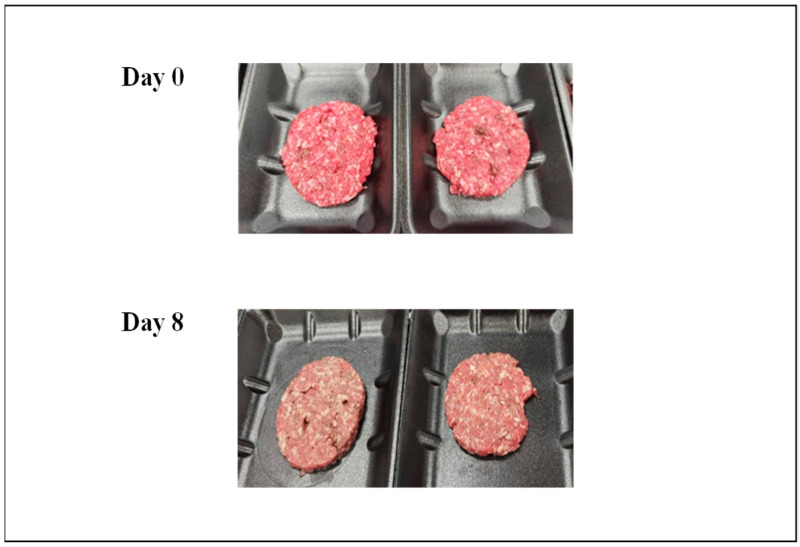
Picture of the addition of goat liver to goat minced meat on day 0 and day 8 of storage. Samples represent 8% goat liver added to the goat minced meat.

Twenty-four goat carcasses (average cold carcass weight of 14.2 kg ± 1.7; refrigerated at 2–4 °C) and their livers were sourced 24 h postmortem from a commercial abattoir and transported under refrigerated conditions to the Queensland Animal Science Precinct (QASP, Gatton, Queensland) and stored in a chiller (2–4 °C) before analysis. The carcasses were deboned, and the muscles and trimmings were cut into cubes (2–3 cm^3^) and mixed thoroughly. The 24 livers were also cut into smaller-sized (1.5 cm^3^) cubes and mixed. Mixed samples (goat meat + liver) (n = 150) were generated by mixing randomly selected goat meat cubes with randomly selected goat liver cubes in the proportions of 0, 2, 4, 6, and 8% w/w (n = 6 replicates per mixture) following the protocol shown in Table 1. For example, the 2% w/w mix was created by weighing and combining 20 g of goat liver cubes to 980 g of goat meat cubes and then mincing the mixture. The 1000 g mixtures were individually minced through a 3 mm plate, with the mincer blade, barrel, and plate being cleaned and dried between each mincing activity. The minced mixture samples were formed into 100 g burger patties (5 patties per replicate), and each patty was placed into a polyethylene terephthalate (PET) tray and covered with plastic over-wrap for further storage (Figure 1). The samples (n = 150) were stored for 8 days in the QAAFI Meat Lab’s chiller (QASP, Gatton, Queensland) at 2–4 °C. Samples were taken from the storage and equilibrated for 30 min at room temperature (16 °C), whereafter the colour, pH, and NIR spectra were measured. Samples were removed from the chiller and evaluated on days 0, 2, 4, 6, and 8 before being discarded.

### 2.2. CIELab Parameters and pH Measurements

The pH of the samples was obtained using a handheld pH meter (Hanna HI98163 Hanna HI198163, Victoria 3173, Australia), calibrated using pH 4 and 7 according to the manufacturer’s instructions and recalibrated every six readings [29]. The pH measurements were taken in duplicate. Approximately 30 min after exposing the fresh surface of each sample, CIE L*, a*, and b* light reflectance was measured at room temperature using a Chromameter CR-400/410 (Thermo Fisher Scientific Australia Pty Ltd., 5 Caribbean Drive, Scoresby, VIC, 3179) set at d:0° (diffuse illumination/0° viewing angle; specular component included) with a standard observer angle of CIE: 2° [29]. The Chromameter was placed on a white tile to calibrate as per the supplier’s instructions. The meter was then placed on the sample at optical infinity (minimum thickness of meat 15 mm), and the colour was measured at three different sites on the surface of the sample and then averaged. The average reading values of L*, a*, and b* were recorded and repeated for all mixture samples [29]. The reported red-green spectrum (CIE a*) and blue-yellow spectrum (CIE b*) values were then used to calculate the Hue° (colour definition) and Chroma values (saturation/colour intensity) for each minced sample by using the following equations:

$$Hue-angle\left({}^{\circ}\right)={tan}^{-1}\left(\frac{b^{*}}{a^{*}}\right)$$

$$Chroma\left(C^{*}\right)=\sqrt{\left(a^{*2}+b^{*2}\right)}$$

### 2.3. NIR Spectra Collection

The NIR spectra (950–1600 nm, 10 nm wavelength resolution) of the samples were collected at day 0 and after each day of storage (2, 4, 6, and 8 days) using a portable NIR instrument (Micro-NIR 1700. Viavi, Milpitas, CA, USA). The proprietary software provided by the instrument manufacturer was used for instrument setup and diagnostics, as well as to collect the spectra (MicroNIR Pro 3.1, Viavi, Milpitas, CA, USA). In this study, 50 µs was used as the integration time, while 50 scans were automatically averaged during spectra collection. The spectra collection was achieved by locating the head of the instrument on top of the sample, where three scans were randomly collected. The three spectra were averaged for further analysis. A reference spectrum was collected between samples (every 20 in this study) using a Spectralon^®^ tile, where the head of the instrument was cleaned and dried with a Kimwipe^TM^ between samples.

### 2.4. Data Analysis

The NIR spectra were exported from the instrument software using the MicroNIR Pro (Viavi, Milpitas, CA, USA) format into Vektor Direktor™ (Version 1.1; KAX Group, Sydney, NSW, Australia) for chemometric analysis. The NIR spectra were smoothed and pre-processed using the Savitzky–Golay second derivative (second-order polynomial, number of smoothing points equal to 9) [30]. Principal component analysis (PCA) and partial least squares (PLS) regression were developed using full cross-validation (leave one out) [31]. The PLS regression was used to develop calibration models for the L*, a*, and b* parameters. The PLS algorithm was used to create dummy models to classify samples according to the level of liver addition to the minced meat samples and to predict the mixtures’ storage days. The coefficient of determination in cross-validation (R^2^_cv_) and the standard error in cross-validation (SECV) were used to evaluate the performance of the models to predict days of storage of the patties [31,32]. The total number of samples was n = 150 (5 levels of addition of liver to minced meat × 5 days of storage × 6 samples per treatment). This study used a multivariate strategy to measure spectral distances or differences between samples to select samples for calibration and validation [33,34]. The Kennard–Stone (KS) algorithm is a classic method used for sample selection. The KS algorithm estimates the distance between samples by selecting samples evenly distributed in the predictor space [33,34]. The KS algorithm utilises the Euclidean distance and has been widely applied to select samples in different applications using spectroscopy [33,34]. The samples in the data set were divided into calibration (n = 90) and validation (n = 60) sets using the KS algorithm. The residual predictive deviation (RPD = SD/SECV) value, the standard error of prediction (SEP), and the coefficient of correlation in prediction were used to evaluate the ability of the PLS models to predict these parameters in the validation set of samples [31,32].

The mean values for the CIELab parameters and pH were statistically analysed using ANOVA with the sample as a random effect and treatment and shelf-life day as the main effects. For post hoc testing, Fisher’s Least Significant Difference (LSD) was used for the multiple comparison test, and a 5% probability level was used (*p* ≤ 0.05) to indicate significant differences.

## 3. Results and Discussion

### 3.1. Changes in CIELAB Parameters and pH Due to Liver Addition and Storage

The pH values and CIELab parameters (L*, a*, and b*) measured of the samples stored for up to 8 days are shown in Table 2. No statistical differences were observed in the pH values of the samples associated with either the level of liver addition or days of storage (ranged from 6.27 to 6.29). The CIELab parameters L* (ranged from 47.6 to 49.8), a* (ranged from 11.6 to 14.8), and b* (ranged from 15.7 to 17.7) also did not differ (*p* > 0.05) between samples or over storage time. However, a slight change in pH value, tending to increase from day 0 to day 8 of storage, was noted. The CIELab values were obtained from the five levels of liver addition to minced meat, where the mean value for L* was 49.84, for a* 12.01, and b* 16.32. As the amount of liver added and the number of days of storage increased (Table 2), the colour ordinates (L* and a*) decreased slightly, indicating that the mixed samples became darker; this could be associated with the presence of the darker pieces of liver as well as with the development of metmyoglobin. Table 2 also shows the change in Chroma and Hue° as the amount of liver and storage days increased. There was a significant decrease in the Chroma (Table 2) and an increase in the Hue° (Table 2) values as the amount of liver added to the patties increased. These differences could be attributed to the darker colour of the liver—it is hypothesised that if the liver ratio were to be increased further, this could lead to consumer resistance against the darker-coloured patties.

### 3.2. Changes in the NIR Spectra Due to the Level of Liver and Days of Storage

**Figure 2:**
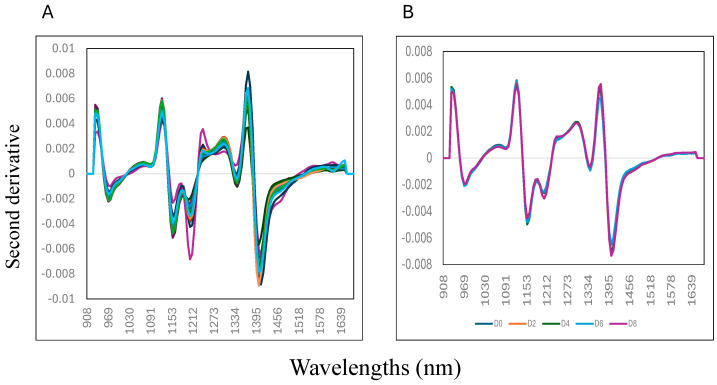
Mean second derivative near-infrared spectra of adding goat liver to goat minced samples: (**A**) mixtures; (**B**) storage.

Figure 2 (panel A) shows the samples’ second derivative mean NIR spectra (0 to 8% w/w) collected on day 8 of storage. Variation in the second derivative NIR spectra of the samples can be observed at five distinctive wavelengths, around 970 nm, 1150 nm, 1217 nm, 1341 nm, and 1403 nm. The mix samples with 0% liver addition showed the highest absorbance at 1440 mm compared to the other samples, where the highest addition of liver (8% w/w) into the mixture showed a lower absorbance value. This wavelength is associated with the O-H bonding, suggesting that changes in moisture or water content of the mixture samples might contribute to explaining the differences between the samples analysed [35,36,37]. It is important to highlight that water is considered a good IR absorber and that the characteristics of water predominate in samples’ NIR spectra, particularly when samples, like meat, have high water levels (>80%). Figure 2 (panel B) shows the second derivative mean NIR spectra of the mixture samples (0 to 8% w/w) collected on each day of storage (from day 0, 2, 4, 6, and 8). It was observed that the samples collected on day 8 of storage had lower absorbance values at each wavelength compared with the samples collected on day 0 (start of the experiment).

### 3.3. PLS Cross-Validation Models for the Measurement of CIELab Parameters

**Figure 3:**
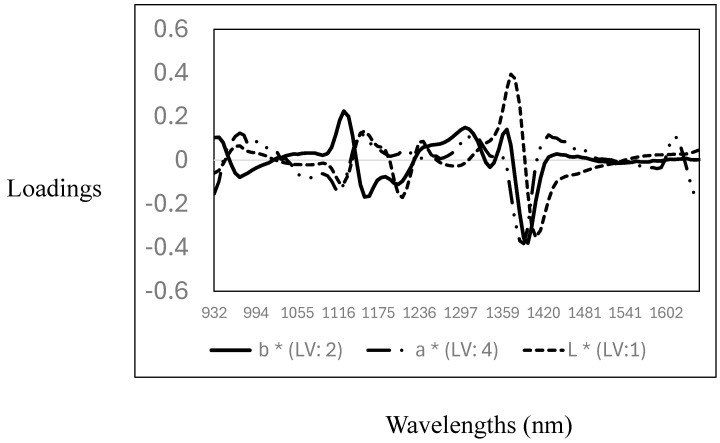
Optimal partial least squares loadings used to predict CIELab parameters (L*, a*, and b*) in the goat mixture samples analysed using near-infrared spectroscopy.

The descriptive statistics (mean, standard deviation, and coefficient of variation), cross-validation, and prediction statistics for the measurement of the L*, a*, and b* parameters using NIR spectroscopy are reported in Table 3. The CV for the CIELab parameters showed that L* (6.7%) and b* (8.5%) have a very low variability, while the highest variability was observed for the a* value (17%). The R^2^_CV_ and SECV were 0.10 (SECV: 3.3), 0.60 (SECV: 1.5), and 0.55 (SECV: 0.90) for L*, a*, and b*, respectively. The RPD values obtained ranged from 1 to 1.5, making the models marginal for quantitatively predicting the CIELab values in the samples analysed. Other authors reported similar results where NIR spectroscopy was used to predict CIELab parameters in pork and poultry meat samples [38,39,40,41]. Furthermore, the PLS calibration models obtained in this study support the data reported in the previous section, indicating that no differences were observed in the CIELab parameters measured. Therefore, the PLS models did not yield acceptable calibrations for the CIELab parameters, as no variability in the data set was observed (see descriptive statistics Table 2). The optimal and highest PLS loadings used for the calibration models are reported in Figure 3. The loadings for the L* values were observed around 1118 nm, associated with C-H, around 1211 nm and 1280 nm, associated with C-H from fat and protein contents, and around 1400 nm with O-H, mainly with both moisture and protein contents. For the prediction of the a* values, the pattern in the PLS loadings was similar to that observed for the prediction of the L* value, with two primary loadings around 1118 nm (C-H) and 1380 nm (C-H), both wavelengths associated with fat and lipid content. For the prediction of the b* values, the optimal and highest loadings were observed around 963 nm (O-H), associated with water or moisture content, and around 1149 nm (C-H), 1211 nm (C-H), 1335 nm, and 1397 nm (C-H), related to fat/lipids and protein content [35,36,37]. These results show a relationship between the CIELab colour ordinates, fat/lipids, and moisture content. This is not surprising, as these parameters are prone to changes (e.g., oxidation) due to the storage of the meat.

### 3.4. PLS Cross-Validation Models for the Prediction of the Addition of Liver and Days of Storage

**Figure 4:**
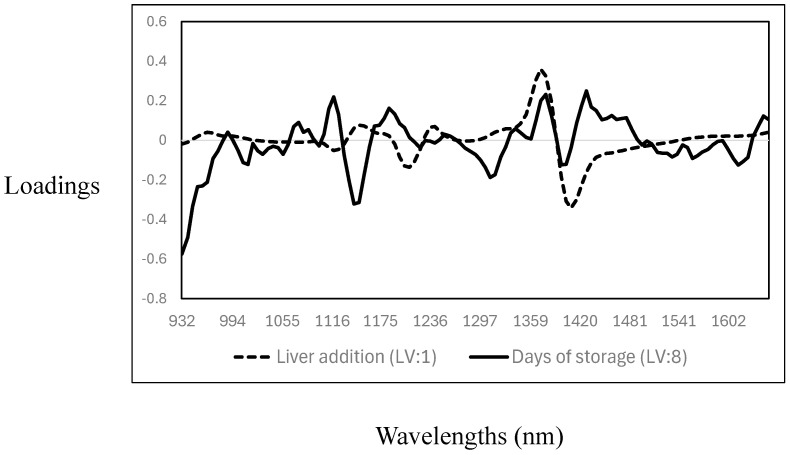
Optimal partial least squares loadings used to predict the level of addition of goat liver to goat minced meat and days of storage.

The cross-validation statistics for the prediction of days of storage and the addition of liver into the meat samples are reported in Table 4. The R^2^_CV_ and SECV were 0.32 (SECV: 2.4%) and 0.92 (SECV: 0.98 days) for the addition of liver (mixing level) and days of storage, respectively. The RPD values indicated that the PLS calibration models could predict the days of storage (RPD > 3); however, no acceptable prediction of the level of liver addition to minced meat was achieved. The poor cross-validation statistics obtained for the prediction of the level of liver added can be explained by the narrow range of liver concentrations tested (0%, 2%, 4%, 6%, and 8%), potential spectral similarity between the liver and muscle tissues, and homogenisation effects that likely contribute to the model’s poor sensitivity. It is important to note that in comparison with the results of this study, most of the previous studies reported on the use of either NIR spectroscopy, MIR spectroscopy, or hyperspectral imaging utilised high proportions of offal addition to minced meat [15,27,28]. Each of the PLS cross-validated calibration models had different and unique loadings, where the number of latent variables (LV) used also differed (liver addition—LV:1; days of storage—LV: 8) (see Figure 4). The highest PLS loadings for the prediction of the level of addition showed only three prominent bands: around 1120 nm and 1205 nm, associated with C-H related to fat content, and around 1403 nm, associated with O-H bonds, mainly related to water [35,36,37]. The highest PLS loadings for the prediction of days of storage were observed around 1007 nm, a wavelength associated with aromatic amines [27], and around 1140 nm (C-H), 1306 nm, and 1354 nm. These wavelengths are associated with C-H related to fat, lipids, and protein contents, while the association around 1403 nm is with O-H (moisture content) [35,36,37]. Other authors reported similar results on using NIR spectroscopy to predict days of storage or shelf life in different meat species [42,43,44,45,46].

The utilisation of techniques such as pH and CIELab, traditionally used by the meat industry alone, does not guarantee the proper identification of the level of addition of offal to minced meat or the evaluation of the days of storage. The appropriate application and implementation of NIR spectroscopy can provide the information required to develop tools for quality assurance and the traceability of meat throughout the meat supply and value chains. To target adulteration and fraud issues, NIR spectroscopy should be combined with other techniques, such as DNA fingerprints, to provide a robust tool to better trace the addition of offal to meat [5,22,47,48]. Near-infrared spectroscopy has been used successfully to collect information about the meat or product’s status and to distinguish meat freshness and refrigeration time [5,22,47,48]. Furthermore, NIR spectroscopy has been adequate in predicting the days of storage of the minced meat and mixtures up to 8 days of storage. Although NIR spectroscopy has the advantage of being fast, non-destructive, accurate, and fit for purpose, issues associated with the required level of data processing and inherent difficulties during calibration development hinder its inclusion as an analytical method by the meat industry [49,50,51].

## 4. Conclusions

The changes in the L*, a*, b*, Hue°, Chroma colour ordinates, and pH showed that the mixtures become darker with an increase in liver addition as well as with the increase in days of storage. The pH values did not change during the storage (days of storage) or due to the level of liver added to the minced meat. The PLS models developed to predict the CEILab parameters were inadequate in predicting these values on new samples quantitatively. Furthermore, the PLS calibration models used to predict the level of liver addition and days of storage gave different results. Although the PLS models are suitable for monitoring shelf life, they do not allow for the detection of low levels of goat liver adulteration. The PLS loadings showed that the PLS algorithm used different regions in the NIR spectra range to develop the calibration models. Compared with traditional methods such as CIELab via a spectrophotometer or pH measurement via a pH meter, NIR spectroscopy can yield results in a much shorter time. However, the variability in the data set should be larger to allow the development of reliable models. Further experiments will be carried out using different levels of addition (e.g., mixtures) as well as including other types of offal (e.g., kidneys), as well as to test other biochemical and chemical parameters related to the shelf life of these type of mixtures. Most meat and meat product enterprises are still accustomed to using traditional methods for detection due to the poor understanding of NIR spectroscopy, including the advantages and limitations of the technology.

## Figures and Tables

**Table 1:** Summary of the treatments and amount and percentage of addition of goat liver to goat minced meat.

Treatment	Goat Meat %	Liver %	Goat Meat (g)	Liver (g)
T0	100	0	1000	0
T2	98	2	980	20
T4	96	4	960	40
T6	94	6	940	60
T8	92	8	920	80

**Table 2:** Mean values and standard deviation of the CIELab (L*, a*, and b*) parameters and pH measured in the mix samples with different levels of addition of goat liver to goat minced meat and storage at days 2, 4, 6, and 8.

Parameter	Days of Storage	% Addition of Goat Liver to Goat Minced Meat				
		0	2	4	6	8
pH	Day 0	6.26 ± 0.01	6.26 ± 0.01	6.29 ± 0.01	6.28 ± 0.01	6.28 ± 0.01
	Day 2	6.27 ± 0.01	6.28 ± 0.01	6.30 ± 0.01	6.30 ± 0.01	6.30 ± 0.01
	Day 4	6.28 ± 0.01	6.27 ± 0.01	6.29 ± 0.01	6.30 ± 0.01	6.30 ± 0.01
	Day 6	6.26 ± 0.01	6.28 ± 0.01	6.29 ± 0.01	6.28 ± 0.01	6.28 ± 0.01
	Day 8	6.26 ± 0.01	6.28 ± 0.01	6.29 ± 0.01	6.29 ± 0.01	6.29 ± 0.01
L*	Day 0	49.7 ± 4.2	49.7 ± 3.2	49.2 ± 3.3	47.8 ± 3.1	48.1 ± 3.5
	Day 2	49.7 ± 4.2	49.5 ± 3.5	49.2 ± 3.6	47.7 ± 3.0	48.0 ± 3.6
	Day 4	49.5 ± 4.4	49.6 ± 3.5	49.1 ± 3.8	47.6 ± 2.9	47.9 ± 3.5
	Day 6	49.3 ± 4.3	49.7 ± 3.5	49.1 ± 3.7	47.4 ± 3.0	47.8 ± 3.5
	Day 8	49.2 ± 4.4	49.7 ± 3.5	48.9 ± 3.7	47.4 ± 3.0	47.8 ± 3.6
a*	Day 0	12.0 ± 2.2	12.0 ± 2.2	12.3 ± 2.2	12.2 ± 2.2	12.1 ± 2.2
	Day 2	14.8 ± 2.2	14.8 ± 1.8	14.8 ± 1.8	14.8 ± 1.8	14.8 ± 1.8
	Day 4	12.9 ± 2.1	12.9 ± 2.2	13.1 ± 2.2	13.1 ± 2.1	13.2 ± 2.2
	Day 6	12.5 ± 1.8	12.4 ± 1.8	12.6 ± 1.8	12.8 ± 1.8	12.7 ± 1.8
	Day 8	11.5 ± 1.8	11.5 ± 1.8	11.6 ± 1.8	11.7 ± 1.8	11.6 ± 1.8
b*	Day 0	16.2 ± 1.3	17.5 ± 1.7	16.2 ± 1.6	16.1 ± 1.3	15.6 ± 1.3
	Day 2	16.3 ± 1.4	17.6 ± 1.7	16.3 ± 1.7	15.9 ± 1.3	15.6 ± 1.4
	Day 4	16.3 ± 1.5	17.6 ± 1.7	16.3 ± 1.7	16.0 ± 1.2	15.6 ± 1.4
	Day 6	16.3 ± 1.5	176 ± 1.7	16.3 ± 1.6	16.1 ± 1.2	15.7 ± 1.3
	Day 8	16.3 ± 1.5	17.7 ± 1.6	16.4 ± 1.6	16.1 ± 1.2	15.7 ± 1.3
Chroma	Day 0	20.2 ± 1.1	22.8 ± 1.1	20.8 ± 1.3	20.5 ± 1.3	20.5 ± 1.3
	Day 2	20.3 ± 1.3	22.9 ± 1.1	20.8 ± 1.5	20.2 ± 1.4	19.4 ± 1.4
	Day 4	20.4 ± 1.2	22.9 ± 1.5	20.9 ± 1.5	20.4 ± 1.2	19.5 ± 1.7
	Day 6	20.3 ± 1.3	23.1 ± 1.2	20.9 ± 1.5	20.5 ± 1.3	19.5 ± 1.8
	Day 8	20.4 ± 1.3	23.1 ± 1.3	21.1 ± 1.3	20.5 ± 1.4	19.6 ± 1.3
Hue°	Day 0	43.5 ± 1.3	45.6 ± 1.3	42.8 ± 1.3	41.2 ± 1.3	42.6 ± 1.3
	Day 2	25.3 ± 1.3	24.9 ± 1.3	30.3 ± 1.4	32.9 ± 1.4	46.8 ± 1.4
	Day 4	30.7 ± 1.3	24.9 ± 1.3	30.4 ± 1.6	31.7 ± 1.5	43.9 ± 1.4
	Day 6	33.4 ± 1.3	24.9 ± 1.5	30.0 ± 1.6	30.8 ± 1.5	42.5 ± 1.4
	Day 8	48.0 ± 1.3	25.2 ± 1.4	29.4 ± 1.5	31.4 ± 1.4	43.6 ± 1.4

Brightness (L*), redness (a*), and yellowness (b*); ±standard deviation.

**Table 3:** Descriptive, cross-validation, and validation statistics for predicting CIELab parameters in the mixture samples analysed using near-infrared spectroscopy.

	n	Mean	SD	Range	SECV	R^2^_CV_	RPD
CAL	90						
L*		48.9	3.3	59.2–39.8	3.3	0.10	1.0
a*		12.9	2.2	17.1–7.6	1.5	0.63	1.5
b*		16.4	1.4	20.3–13.1	0.90	0.60	1.6
VAL	60				SEP	R	
L*		48.7	2.9	56.9–41.6	2.9	0.10	
a*		13.1	1.86	17.2–8.8	1.3	0.58	
b*		16.7	1.3	20.4–14.5	1.5	0.73	

CAL: calibration; n: number of samples; SD: standard deviation; SECV: standard error of cross-validation or standard error in prediction; R^2^_cv_: coefficient of determination in cross-validation; R: coefficient of correlation in prediction; RPD: residual predictive deviation; VAL: validation; brightness (L*), redness (a*), and yellowness (b*).

**Table 4:** Cross-validation and validation statistics for predicting the level of addition of goat liver to minced meat and days of storage in the samples analysed using near-infrared spectroscopy.

	SECV	R^2^_cv_	RPD	SEP
CAL—level of addition (n:90)	2.4	0.32	1.0	
VAL—level of addition (n:60)		0.23		3.1
CAL—days of storage (n = 90)	0.98	0.92	2.8	
VAL—days of storage (n = 60)		0.90		1.12

CAL: calibration; n: number of samples; SD: standard deviation; SECV: standard error of cross-validation or standard error in prediction; R^2^_cv_: coefficient of determination in cross-validation; RPD: residual predictive deviation; VAL: validation.

## Data Availability

The original contributions presented in the study are included in the article, further inquiries can be directed to the corresponding author.
